# Pregnancy Burden: An Integrative Review and Dimensional Analysis of Pregnancy's Hidden Challenges

**DOI:** 10.1111/jmwh.13759

**Published:** 2025-05-22

**Authors:** Hannah E. Kumarasamy, Felesia Bowen, Becca Billings, Patricia A. Patrician

**Affiliations:** ^1^ University of Alabama, School of Nursing Birmingham Alabama; ^2^ Department of Acute, Chronic & Continuing Care University of Alabama, School of Nursing Birmingham Alabama; ^3^ Lister Hill Library Department of Clinical, Academic, & Research Engagement University of Alabama Birmingham Birmingham Alabama; ^4^ Department of Family, Community & Health Systems University of Alabama, School of Nursing Birmingham Alabama

**Keywords:** determinants of health, health equity, hidden burden, pregnancy, perinatal health, pregnancy burden

## Abstract

**Introduction:**

Outcomes surrounding childbirth have focused on survival, leaving gaps in understanding the comprehensive experience of pregnancy for the pregnant individual. Anecdotally, pregnancy and the opportunity to reproduce is often received with a celebratory response. Yet whether planned or unplanned, a wide array of burdens may exist throughout pregnancy ranging from minor inconveniences to dangerous contributions to morbidity and mortality. The experience of pregnancy is superimposed onto the physical, mental, and social reality that already exists as an individual's life and consistently accentuates aspects of stress that can lead to increased physical, mental, emotional, financial, or other burden that many health and social systems globally lack resources to support. To address this gap, this analysis sought to explore the concept of pregnancy burden.

**Methods:**

A formal search of 5 databases was conducted using integrative review methodology, with a total of 37 articles meeting inclusion criteria. To better conceptualize pregnancy burden, a dimensional analysis was then undertaken posing the research question, “What is pregnancy burden?”

**Results:**

The current social construction of pregnancy burden revealed multidimensional contributors to burden that were identified as both intrinsic and extrinsic, with no current definition available. Five dimensions of pregnancy burden were discovered: health, education, financial or cost, inequity, and social support. Three distinct perspectives were identified that included the pregnant person; their partners, family, or friends; and health systems or care providers. To best answer the research question and focus on the personal experience, the scope of this analysis was limited to the perspective of the pregnant individual.

**Discussion:**

The term *burden* is discussed and well‐developed in chronic disease literature but has not been inclusive of pregnancy. This review revealed that pregnancy burden exists but remains unclassified and understudied, supporting the need for further exploration. Better understanding and valuing of the total experience of pregnancy, inclusive of burden, has the potential to improve the pregnancy experience.

## INTRODUCTION

Birth outcomes have largely focused on the physical aspects of pregnancy centered in survival, leaving gaps in appreciating the comprehensive nature of the experience for the pregnant individual.^1^, ^2^ As such, significant research within the current body of maternal health literature has focused on increasing knowledge of pathologic conditions associated with pregnancy and less on normal physiologic processes. What remains understudied, however, is the multifaceted burden that accompanies a normal physiologic pregnancy experience and how this burden might be affected by the scaffolding effects of a pathologic condition, social and structural factors, or personal circumstances.^3^, ^4^ Improving understanding of both the physiology of pregnancy and the pathology of unexpected conditions allows for the development of care and treatment guidelines for providers but may only be useful in improving outcomes to the extent that the experience and needs of the pregnant individual are recognized and addressed.^3^, ^4^, ^5^, ^6^ The influence of pregnancy burden as a holistic measure of pregnancy‐related factors, experiences, and needs remains undefined and thus unmeasured.

**QUICK POINTS**

✦ The physiologic experience of pregnancy is intrinsically accompanied by an array of burdens that may be simply described as a nuisance or more advanced in complexity and lead to extreme compromise in capacity that impacts safety and maternal health outcomes.
✦ The concept of pregnancy burden remains understudied but may encompass and describe a myriad of multilevel factors that accumulate and are associated with the entire pregnancy experience for the individual.
✦ Further exploration and development of the concept of pregnancy burden could allow for advancement of clinical care, social systems, and policy evolution to support patients, alleviate burden, and improve outcomes through better understanding of contributors, characteristics, and relationships.

Although pregnancy is a physical, mental, psychosocial, and emotional physiologic process and considered a normal part of the life course, it is accompanied by varying types and levels of burden.^1^, ^7^ This unique time lacks a parallel comparison, and addressing burdens specific to pregnancy offers an opportunity to better support the needs of the pregnant individual.^1^, ^8^ Although pregnancy and the opportunity to reproduce is anecdotally perceived as a celebrated event, whether planned or unplanned, a wide array of burdens may exist, ranging from minor inconveniences to dangerous contributions to mortality and morbidity. Pregnancy has often been viewed as an event rather than part of the life continuum, yet these burdens may have lasting impact.^8^, ^9^ The experience of pregnancy is layered onto the already present physical, mental, and social reality that exists as an individual's life and consistently accentuates aspects of stress that can lead to increased physical, mental, emotional, financial, or other burdens that many health and social systems globally lack resources to support.^10^

The contributors to and impacts of short‐ and long‐term pregnancy burden faced by pregnant individuals are not well defined but appear to influence health for decades after the pregnancy has concluded.^11^ Previous studies have provided evidence of the complex structural and social upstream issues that have been associated with maternal morbidity and mortality and increased burden for the individual.^12^, ^13^, ^14^ Yet the baseline demands of pregnancy are largely unaccounted for, as holistically understood within the context of the pregnant individual's environment, situation, and lived experience. The impact of stress on physiologic functioning has been previously established, but the impact of chronic weathering from discrimination and racism, alongside stress, as a contributor to pregnancy burden is also unclear.^9^, ^11^, ^12^, ^13^, ^14^ Exploring pregnancy burden therefore may offer future opportunities for targeted interventions aimed at addressing these various influencing factors.

For future research to begin to address pregnancy burden, a foundational understanding is necessary to establish universal use and reduce ambiguity in meaning. An opportunity exists to shift the focus of burden in health care from something that an individual places on another outwardly to something that is additionally experienced personally, inward, and of equal importance. This begins by exploring the concept of pregnancy burden, solidifying a literature‐derived theoretical definition, elucidating dimensions, and clarifying perspectives to inform a framework and guide research. Therefore, the aim of this concept analysis is to explore the dimensions and perspectives of pregnancy burden and to define burden in the context of pregnancy.

## METHODS

The dimensional analysis methodology developed by Schatzman, and later modified by Caron and Bowers, was chosen to evaluate the concept of pregnancy burden.^15^, ^16^ This type of analysis was selected because it fits with the many dimensions that exist and the complexity of the spectrum of burden that is experienced in pregnancy.^16^

A dimensional analysis varies from a traditional concept analysis in 4 specific ways: (1) it focuses on construction of the concept and its use, (2) varied perspectives are identified including those not represented, (3) contextual relationships of concepts and assumptions are considered, and (4) presentation of the concept itself, through an exploration of dimensions and perspectives, is relevant in determining the societal and social perspective.^15^, ^16^

Hendrickson and McCorkle (2008) clarified that this form of analysis explicated the dimensions that lead to a construct's development and utilization.^17^ For a new concept, this was determined to be important for a comprehensive review. This method has 4 discrete steps that have shown to support the development of knowledge and theory: (1) describe the concept's social construction, (2) describe the logic of the concept from multiple perspectives and multiple contexts, (3) differentiate the relationship between perspectives and use or definition of the concept, (4) identify and examine assumptions.^15^

A health sciences librarian specializing in systematic reviews (B.B.) collaborated with the authors to develop sensitive and replicable search strategies for the following databases: CINAHL Plus with Full Text (CINAHL), PsycINFO, PubMed, and Scopus. Database‐specific subject headings including CINAHL Headings and Medical Subject Headings were used in combination with keywords specific to pregnancy burden. Concepts covered included (1) pregnancy *(“pregnancy” [subject heading] OR “fertility” [subject heading] OR “family planning” [subject heading] OR “reproductive behavior” [subject heading] OR maternal OR infertility OR prenatal OR postnatal OR postnatal OR perinatal [keywords])* and (2) burden (burden* [keyword]). Additional synonyms for *burden* were not included to retrieve only literature with the specific phrase(s) of *pregnancy burden* or *burdens of pregnancy* or *reproduction*. No limits were applied. The search took place on August 1, 2023. Exact search strategies used for each database are included in Supporting Information: Appendix S1.

After the search string was confirmed, identified articles were uploaded into Covidence for formal review. Inclusion criteria were restricted to articles published in English; PDF full‐text available; human studies; inclusion of individuals who were pregnant or within 1‐year postpartum; relevant quantitative, qualitative, and mixed‐methods research articles or systematic reviews; and relevance to defining, understanding, or an outcome of pregnancy burden. Exclusion criteria included animal studies, sole focus of preconception, not available in English or English translation, greater than 1‐year postpartum, editorials or nonresearch articles, articles presenting future studies but without results, articles without a direct or indirect focus on pregnancy burden, and articles deemed not applicable to establishing knowledge of pregnancy burden. Inclusion criteria were not restricted to a specific timeframe, with the goal of not limiting the context of these search terms to a specific period.

Titles and abstracts were first screened by the lead author to determine which articles met criteria for inclusion. Full text of included articles were then reviewed in their entirety by a subject matter expert (H.E.K.), and screening guidance and agreement was provided by faculty (F.B., P.A.P.). Authors then moved into the analysis phase to identify themes and dimensions. Descriptors of pregnancy burden found in the literature were compiled to determine the social construction of pregnancy burden and consolidated into overarching dimensions. Perspectives, the relationships between dimensions, and the variation of perspectives across dimensions were identified. Subdimensions were uncovered and created further clarity around existing assumptions and the concept. This analysis was completed May 31, 2024.

### Positionality Statement

The authors recognize how identities, background, and privilege shape perspective and influence research and acknowledge the inherent structural advantages of specific groups. The first author identifies as a White, cisgender female, and first‐generation college student raised in an economically and clinically disadvantaged rural farming community. She additionally has the lens of a nurse‐midwife, practicing in an urban safety net setting. The second author identifies as a cisgender female descendent of enslaved people, first‐generation college student, and war veteran raised in a lower‐middle‐class military family. This author views life through the lens of a pediatric nurse practitioner rooted in health equity and social justice. The fourth author identifies as a White, cisgender, first‐generation college graduate from an economically disadvantaged background in the northeastern United States. This author brings forward the lens of a nurse scientist, military veteran, and health services researcher with the lived experience of both pregnancy joy and burden.

## RESULTS

**Figure 1:**
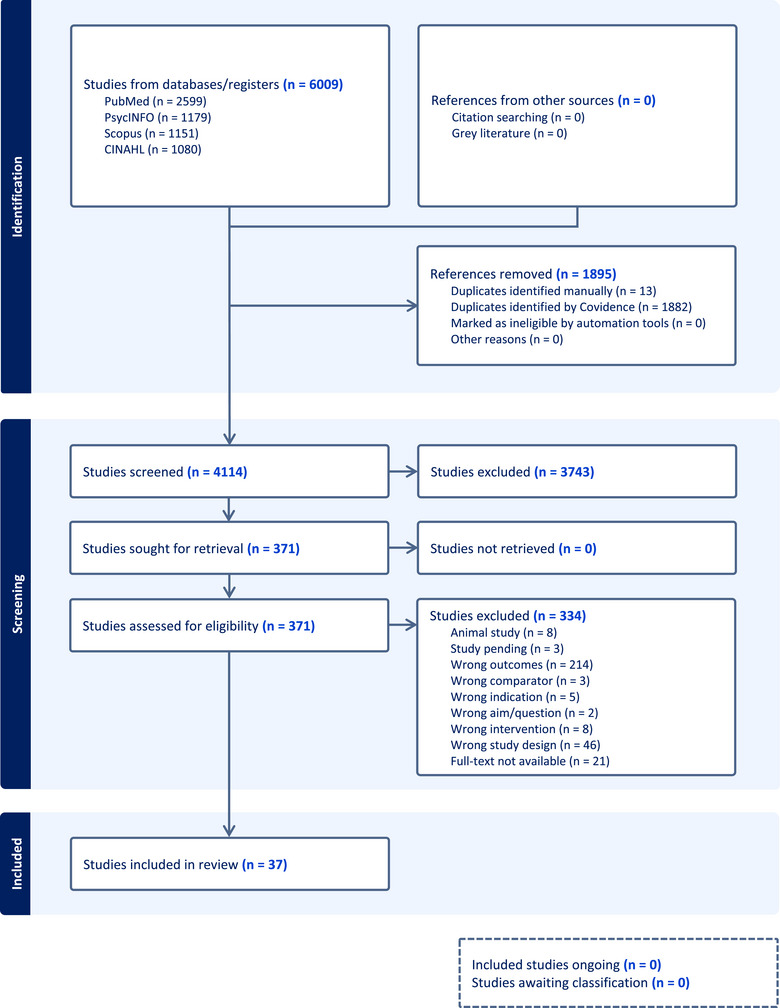
Preferred Reporting Items for Systematic reviews and Meta‐Analyses Diagram for Integrative Review of Pregnancy Burden

The literature revealed multilevel contributors to pregnancy burden that go beyond the scope of this article, as individual‐level contributors will be the primary focus. A total of 6009 articles were identified in the search. Once 1895 duplicates were removed, a total of 4114 references remained for the title and abstract screening stage. Inclusion and exclusion criteria were then applied to full‐text screening, which resulted in a total of 37 articles to be included in the analysis. A Preferred Reporting Items for Systematic Reviews and Meta‐Analyses diagram is referenced in Figure 1. The integrative review results were analyzed through a dimensional analysis using NVivo 12 software to code themes and identify dimensions. The included articles included publications from 5 continents, representing 19 countries (Table 1) of all economic and health status classification levels. Although the breadth of the global results was unexpected, the diversity of contexts aided in cultivating a broader understanding of the social construction of pregnancy burden. The multidimensional contributors to burden were both intrinsic and extrinsic, yet no current definition was found. Pregnancy burden was conceptualized not as negative or positive but as reflective of part of pregnancy experienced by the pregnant individual. The literature matrix displayed in Table 1 provides an in‐depth review of the analysis.

**Table 1:** Associated Dimensions and Terminology of Included Articles

Author (Year)	Sample	Location	Associated Dimensions	Terms Used
Abdollahpour et al (2022)^32^	11 near‐miss women	Iran	Health	Long‐term pregnancy burden
Acharya et al (2016)^54^	384 postpartum women and partner	Nepal	Financial or cost, inequity, social support	Economic burden for pregnant women
Agampodi et al (2021)^48^	n = 233 pregnant adolescents; n = 1037 primiparous individuals from original sample of 3367	Sri Lanka	Education, health, financial or cost, inequity, social support	Hidden burden of adolescent pregnancies
Aung et al (2018)^27^	Adolescents, aged 10‐19, who were pregnant or had given birth	Thailand	Financial or cost, health, inequity	Burden of adolescent pregnancy; burden of maternal disorders
Bloch et al (2018)^20^	Case vignette and GIS‐geocoded maps	United States (Philadelphia, PA)	Financial or cost, health, inequity, social support	Mothers’ burden
Bucher‐Koenen et al (2020)^39^	404,286 mothers aged 55‐65, Swedish residents in 1990	Sweden	Education, financial or cost, health, inequity	Burden on mothers’ health, double burden, burden on women
Callaghan et al (2015)^29^	H1N1 pregnancy‐related deaths between April 2009 and June 2010	United States	Health, inequity	Burden of morbidity and mortality on pregnant women
Callander et al (2021)^55^	Antenatal care recipients at a publicly funded Australian tertiary hospital from October 2016 to August 2018	Australia	Financial or cost, health, inequity, social support	Financial burden of intimate partner violence
Cohen et al (1926, 1927)^18^, ^19^	3000 low‐risk pregnant individuals, with serial examinations	United States (Boston, MA)	Health	Circulatory burden in pregnancy
Doat et al (2022)^51^	Systematic review of 12 primary research studies	Sub‐Saharan Africa	Education, health, financial or cost, inequity, social support	Pregnancy as a burden
Dyer et al (2022)^24^	Maternal deaths in Louisiana, from 2016‐2017 (N = 112)	United States (Louisiana)	Education, health, financial or cost, inequity, social support	Burdensome risk of adverse health outcomes
Gaym et al (2011)^43^	112 hospitals and 685 health centers	Ethiopia	Health, inequity, social support	Maternal disease burden
Geeganage et al (2023)^33^	2006 database: 26 million cases from 955 hospitals in 24 states; 2014 database: 31 million cases from 945 hospitals in 34 states	United States	Health, financial or cost, social support	Burden of hyperemesis gravidarum
Gompers et al (2023)^42^	944 individuals from a larger study; 47.6% completed the study prenatally and 52.4% completed it postpartum	Cross‐sectional survey	Financial or cost, health, inequity, social support	Financial burden of pregnancy
Hagaman et al (2019)^25^	960 women interviewed 4 times over a 15‐mo period	Pakistan	Education, health, financial or cost, inequity, social support	Burden of disability
Huang et al (2022)^31^	Data from Global Burden of Disease 2017 analysis	Global	Health, inequity	Maternal disorder disease burden
Huynh et al (2013)^37^	PubMed search from January 2000 to December 2012, with 40 included articles	United States	Financial or cost, health	Economic burden of pregnancy
Jonsdottir et al (2020)^40^	Longitudinal data from 503 women	Iceland	Health, financial or cost, inequity	Burdening condition for pregnant women
Knittel et al (2023)^23^	34 interviews of 38 jail employees	United States (5 states: AL, GA, NC, SC, WV)	Health, financial or cost, inequity, social support	Burden due to pregnancy
Kourlaba et al (2016)^30^	Systematic review of 20 articles that met inclusion criteria	Systematic review and meta‐analysis	Financial or cost, health, inequity	Humanistic and economic burden of pregnancy‐related VTE
Lambers et al (2021)^50^	All patients who birthed triplets and their neonates between April 2007 and October 2014; pregnant individuals n = 82, neonates n = 246	United States (Cincinnati, OH)	Health, financial or cost, inequity, social support	Maternal health burden
Law et al (2015)^52^	Pregnant women aged 15‐49, totaling 322,141 eligible live births	United States	Financial or cost, inequity	Economic burden of pregnancy in the United States
Leone et al (2013)^22^	9643 households receiving maternal health care in the past year	India	Financial or cost, inequity, education, social support	Relative burden of maternal health care
MacDonald et al (2022)^47^	11 female urologists with academic appointments	United States	Education, financial cost, inequity, social support	Burden of childbearing (including pregnancy/maternity leave)
Marti‐Castaner et al (2022)^21^	RCT participants were invited if they were within 1 year postpartum (N = 20)	United States (New York, NY)	Education, financial cost, inequity, social support	Financial burden; mental burden; “I don't want to be a burden”
Mogos et al (2016)^36^	Birth‐related discharges with a diagnosis code for IPV; N = 3649	United States	Financial or cost, health, social support	Significant health burden
Mousa et al (2022)^45^	Study of N = 83 admitted 2019‐2021 with a diagnosis of preeclampsia	Saudi Arabia (Jeddah)	Health	Pregnancy burden
Nakamura et al (2022)^38^	Pregnant (n = 450) and nonpregnant (n = 454) individuals (total n = 904)	Japan	Education, health, financial or cost, inequity, social support	The physical burden associated with pregnancy
Njim et al (2017)^49^	N = 886 participants	Cameroon	Health, social/system support	The burden of pregnancy
Pedersen et al (2011)^56^	The Danish National Birth Cohort was used; N = 37,127	Denmark	Health, financial or cost, inequity, social support	Psychosocial burdens; felt burdened by their “economy,” “housing situation,” or “job” while pregnant
Piwko et al (2007)^53^	Participants recruited from a telephone support line; N = 149	Canada	Health, financial or cost, inequity, social support	Pregnancy imposes an economic burden
Sharma et al (2022)^46^	Women aged 18‐49 in the National Health Interview Survey; N = 1433	United States	Health, financial or cost, inequity, social support	SDOH burden; financial burden
Shin et al (2020)^34^	N = 1170 Korean physicians	Korea	Education, financial cost, inequity, social support	The burden of pregnancy
Smith et al (2000)^41^	N = 593 individuals presenting with first trimester nausea and vomiting	Australia	Health, financial or cost, social support	Burden of early pregnancy
Thapa et al (2021)^28^	Pregnant women birthing in a tertiary care hospital; N = 1415	Nepal	Health, inequity, social support	The burden of pregnancy induced hypertension
Tinago et al (2018)^26^	Adolescent girls and young women aged 14‐24 (n = 48)	Zimbabwe (Harare)	Education, health, financial or cost, inequity, social support	Pregnancy burden
Zafar et al (2015)^44^	Two rural communities; n = 3459 (Pakistan, n = 1727; Malawi, n = 1732)	Malawi and Pakistan	Health, social support	The burden of maternal morbidity; global burden of pregnancy associated disease

Abbreviations: AL, Alabama; GA, Georgia; IPV, intimate partner violence; MA, Massachusetts; NC, North Carolina; NY, New York; OH, Ohio; PA, Pennsylvania; RCT, randomized controlled trial; SC, South Carolina; SDOH, social determinants of health; VTE, venous thromboembolism; WV, West Virginia.

### Dimensions and Perspectives

Dimensions are attributes of the concept that provide context and illuminate relevant perspectives central to the meaning of the construct, to support defining the concept.^15^ Five dimensions emerged through this analysis of pregnancy burden: health, education, financial or cost, inequity, and social support. Within each dimension are relevant perspectives that describe the logical aspects of the construct to create meaning and consider varying contexts.^15^

Three perspectives were evident and included the pregnant person; partners, family, or friends; and health systems or care providers. Pregnant individuals interact with and are vulnerable to the health care system and care providers, whereas partners, family, or support persons are generally interacting with the pregnant person and not the system. The influential nature of the dynamics between perspectives and the importance of support systems was discussed throughout the literature. The dimensions are further broken into subdimensions, and the context of the dimension or subdimension's role often varies by perspective. This article explicitly focuses on the perspective of the individual; however, further details surrounding the other perspectives are outlined in Table 2 and are beyond the scope of this article.

**Table 2:** Summary of Dimensions and Attributes by Perspective

Dimension by Perspective	Pregnant Individual	Health System/Care Providers	Partner/Family/Support
Health	Face preconception barriers to accessing care^20^	Limitations to optimizing health prior to pregnancy^26^	Feel providers inadequately communicate with family^32^
	Impacted by multiple factors outside of their control^20^, ^21^, ^23^, ^24^, ^25^	Constraints in time, policy, and counseling^26^	Focused on supporting healing of pregnant individuals, including mentally and emotionally^32^
	Frequently have limited knowledge of physiologic adaptation of pregnancy and postpartum that will occur and how this impacts health^26^	Risks of conception are often not conveyed until after conception occurs^27^	
	Intrinsic normal physiologic changes occur physically with conception^18^, ^19^	Goal of optimizing physical outcomes^32^	
	Experience pregnancy as a mental and emotional experience as well^27^	Outcomes viewed in terms of survival^24^, ^27^, ^45^, ^46^	
	Health and burden have a bidirectional impact^20^, ^33^, ^36^, ^40^, ^41^, ^42^, ^43^, ^44^		
Education	Higher education levels increase access to resources, including family planning^25^, ^34^, ^37^, ^47^, ^51^	Limitations globally around support structures for adolescents who become pregnant and after birth^27^, ^48^, ^49^	Essential in navigating pregnancy and educational opportunities/advancement^21^
	Education does not mitigate effects of structural and systemic racism^23^, ^24^, ^46^	Family planning services often less accessible to individuals with less resources^20^, ^21^, ^42^	Access to positive social support systems was higher in individuals with higher education levels^25^, ^34^, ^47^
	Risk of not completing high school for adolescents who become pregnant^27^, ^48^, ^49^	View of cost burden from unintended pregnant, with limited solutions^37^	
	Although educational advancement may increase income and resource access, immediate financial needs must often be prioritized—especially for individuals living in poverty^21^		
	Returning to education/training posed significant burden in all socioeconomic groups^21^, ^34^, ^38^, ^47^		
	Negotiating professional and person priorities for pregnancy^34^, ^37^, ^39^, ^47^, ^50^		
Financial or cost	Increased time and financial investment during high‐risk pregnancy^20^	Care costs and expenditure continue to increase in many countries^22^, ^37^, ^52^, ^53^	Adolescent pregnancies globally often place financial strain on families^26^, ^27^, ^48^, ^49^
	Out‐of‐pocket costs for care may prohibit receiving care and increases risk for greater health and financial burden^37^	National health care programs for pregnant individuals vary widely between countries, with care costs often prohibiting access^37^	May offer financial, emotional, and opportunity cost support^21^, ^42^, ^47^
	Navigating multiple competing demands, perpetuating economic inequality over time^20^, ^21^, ^47^	Systems and support globally may impose irreversible opportunity cost^21^, ^47^	
	Pregnancy intention and socioeconomic status may impact long and short‐term stability^20^, ^21^		
	Stability is multifactorial and affected by health, employment, benefits, social support, safety, and conflict^21^, ^36^, ^37^, ^38^, ^40^, ^42^		
	Risk to long‐term risk to health with morbidity^44^, ^45^		
Inequity	Global experience of gender discrimination.^32^	Reliance on women and pregnant people as inherent solutions for unmet needs^20^, ^21^, ^22^, ^23^, ^34^, ^39^, ^47^	Level of support impacts the pregnant individual's ability to meet their own needs versus always caring for others^21^
	Gendered expectations creating and perpetuating hidden burden^18^, ^20^, ^21^, ^22^, ^24^, ^25^, ^26^, ^27^, ^30^, ^31^, ^33^, ^34^, ^36^, ^37^, ^38^, ^39^, ^40^, ^41^, ^42^, ^43^, ^45^, ^46^, ^47^, ^48^, ^49^, ^50^, ^52^, ^53^, ^54^, ^56^	Expectations consistent among all income levels, with differing responsibilities and demands^21^, ^34^, ^47^	
	Family “case manager,” often at the expense of their needs being unacknowledged^20^		
	Racial inequity is layered onto gender discrimination, impacting multiple levels of lived experience, including health and care received and outcomes^21^, ^23^, ^24^, ^37^, ^42^		
Social support	Factors pertaining to the social determinants of health positively and negatively impact burden experienced and may impede ability to access resources necessary to be successful^21^	Social safety net and health system access often creates barriers to improving circumstances^20^, ^21^	Essential in moderating and managing pregnancy burden^24^, ^40^, ^41^, ^53^, ^56^
	Extra support is needed during pregnancy and postpartum, and when needs are unmet, this time may contribute to further instability^21^	Often accompanied by unrealistic expectations to navigate^21^	Positive spousal support impacts maternal success^40^, ^47^
	Need satisfaction in relationships and support systems^38^, ^40^, ^47^		Strong family support influences adjustment to maternal role, outcomes, income, and childcare^21^, ^34^, ^40^, ^47^
	Navigate limitations system support of career, childcare, health care, housing, etc, during pregnancy and beyond^20^, ^21^		Relationship dissatisfaction or limited family support contributed negatively to outcomes and life^38^, ^40^, ^47^
			May increase pressure for pregnancy^51^

Throughout the analysis, physiologic changes to body systems required to accommodate pregnancy (hemodynamic, hormonal, cardiac, renal, metabolism, etc), were ubiquitous to increased intrinsic pregnancy burden.^18^, ^19^ Not all factors contributing to pregnancy burden were intrinsic, however. Many extrinsic factors notably had a significant effect on pregnancy burden and influenced or were influenced by the discovered dimensions.

To provide greater insight into the dynamic interplay discovered between key aspects of the dimensions and the experience of pregnancy burden, the subsequent sections will provide further detail into each dimension and subdimension.

### Health

This dimension included the subdimensions of *physical* and *mental or emotional*. Optimizing health prior to conception prepares the body to physically compensate for the physiologic changes of pregnancy, but barriers to care frequently exist globally.^20^, ^21^, ^22^ Systemic and structural factors, such a discrimination and racism,^21^, ^23^, ^24^ and factors influencing the social and structural determinants of health^20^, ^21^ further influence access to resources,^20^, ^23^ baseline health and wellness,^25^ and lived experience^21^ prior to, during, and after pregnancy, which all further contribute to the relationship between pregnancy burden and health.

#### Physical

The oldest conceptualization of pregnancy burden found in the literature was first discussed in 1936.^18^ This work described dynamic cardiovascular changes inherent to pregnancy, which place physical burden on the pregnant individual.^18^, ^19^ Although baseline risk may alter the level of cardiovascular burden experienced, changes within the cardiovascular system to accommodate pregnancy are unavoidable.^18^, ^19^ Individuals with known pathologic conditions that may worsen with pregnancy (eg, cardiovascular conditions) often receive limited counseling prior to conception due to limitations to primary care and family planning access, policy, and evidence‐based counseling constraints.^23^, ^24^, ^26^, ^27^, ^28^

Physiologic changes occur to support the physical accommodation of pregnancy, but impaired compensation can lead to pathologic changes and include unexpected direct or indirect causes of morbidity and mortality.^27^, ^29^, ^30^, ^31^, ^32^, ^33^ Examples of indirect physical burden include the implications of preexisting conditions such as chronic hypertension or diabetes,^23^, ^34^, ^35^ diagnosed or undiagnosed, and infectious disease risks such as influenza pandemics.^27^ Experiences of postpartum hemorrhage or amniotic fluid embolism describe health outcomes that were a direct physical burden.^36^, ^37^

#### Mental or Emotional

Direct and indirect pregnancy burden extended to mental and emotional aspects of health and were frequently impacted by surrounding experiences.^31^, ^33^ After a new diagnosis or health experience, providers and health systems prioritized physical management and individuals closest to the pregnant person reported inadequate communication.^27^ The pregnant individual and their support systems focused on mental health, and healing physically and emotionally, with often limited comprehensive resources available.^27^ Additionally, the relationship between outcomes and pregnancy burden extended past the window of gestation and influenced long‐term morbidity and mortality.^38^, ^39^

The pregnant individuals’ emotional experience may be driven by the level of pregnancy burden experienced at a given time, whereas the experience of health simultaneously impacts the level of perceived burden. Wellness and health perception influenced pregnancy through the experience and management of physiologic symptoms,^33^, ^40^, ^41^ mental health,^40^, ^42^ and access to recommended health promotion activities^20^, ^36^, ^40^, ^43^ but often declined at different points in the pregnancy and postpartum periods.^40^, ^44^ Providers and health systems quantify pregnancy outcomes by measures of survival such as maternal mortality, maternal near‐miss, and severe maternal morbidity,^24^, ^27^, ^45^, ^46^ but this focus excludes pregnancy burden outside of pathologic conditions and limits the acknowledgment of the simultaneously mental and emotional experience of all the reproductive phases.^27^

### Education

The dimension of education was extrinsically influenced and formed by 2 subdimensions, protective factors and family planning. Higher levels of education consistently serve as protective factors, alleviating barriers that contribute to greater extrinsic pregnancy burden, such as access to housing, childcare, food, transportation, and positive social support.^25^, ^34^, ^47^ Despite this, education has notably been unable to moderate the risk imposed by structural inequities, such as racism and discrimination.^25^, ^34^, ^47^ Black women with higher education levels in the United States continue to experience dramatically worse mortality rates than their White counterparts.^23^, ^24^, ^46^

#### Protective Factors

Adolescent pregnancies created barriers to completing high school education and beyond,^27^, ^48^, ^49^ whereas mothers living at or below the poverty level who desired to continue their education were required to prioritize immediate financial needs through opportunities with limited advancement potential.^21^ A qualitative study participant stated,

I got into nursing school, but I can't go because I don't have anywhere to live. Not even that, I don't have nobody to watch him (infant) if I need time to study or watch him in the morning…I wish I had the supports to where I could have went to school and started my career. That way we don't have to go through this anymore.^21^

The struggle of returning to education and training during and after pregnancy was burdensome in all socioeconomic groups,^21^, ^34^, ^38^, ^47^ requiring negotiation of different priorities to accommodate pregnancy.^34^, ^38^, ^39^, ^47^, ^50^ Participants in a retrospective, longitudinal study, with higher levels of education and income and experienced an unplanned twin pregnancy, were found to experience higher mortality rates later in life than individuals with average income and education.^39^ Unexpected twin pregnancy acted as a control for an unintended birth.^39^ The stress of intrinsic and other extrinsic pregnancy burden,^39^ combined with intensive professional requirements, appears to have a relationship with long‐term health.^38^, ^39^, ^47^, ^50^

#### Family Planning

Family planning services were more widely accessed and accessible to groups with higher educational attainment, increasing autonomy over pregnancy timing for individuals with more resources.^37^, ^51^ Yet, the combination of less education and unplanned pregnancy increased vulnerability to fundamental upstream factors such as lower socioeconomic status and limitations to health care access, largely due to differences in financial opportunity.^20^, ^21^, ^42^ Unintended pregnancy was highlighted by health systems as an increased cost burden on the health system, with higher clinical burden, and financial expenditure increasing consistently over the past 3 to 4 decades.^30^, ^46^ This health systems perspective may increase limitations to necessary resources, placing further burden on the pregnant individual if pregnancy is not deemed as worthy of expenditure.

### Financial or Costs

The financial or cost dimension arose throughout the analysis and was largely driven by extrinsic factors, which appear to increase burden experienced by the individual. The subdimensions included short term and long term. Cost notably extended past finances, displaying aspects of trade‐off that pregnant individuals must navigate and negotiate to survive and succeed despite burden imposed by systems.

#### Short Term

Pregnancy and postpartum may foster short‐term costs with the imposition of increased time and financial investment required to meet care recommendations and greater burden in higher‐risk pregnancies.^20^ Despite birth rates decreasing in the United States, pregnancy expenditure has continued to increase, placing further financial burden on individuals.^22^, ^37^, ^52^, ^53^ In countries with limited national support for care, the out‐of‐pocket cost of prenatal care is often prohibitive, which inadvertently increases risk for pregnancy‐related complications and greater short‐ and long‐term health risk and financial burden for the individual.^37^ The short‐term cost of competing demands such as work hours, income, childcare, and other financial commitments increased burden experienced and translated to economic inequality over the long term.^37^

#### Long Term

The cost of an unplanned pregnancy to someone already living in deep poverty further accentuated instability in the short and long term.^20^, ^21^ This type of instability was perpetuated by health problems,^21^, ^38^ underemployment, job loss, lack of employment benefits,^21^, ^42^ interpersonal conflicts,^21^, ^36^ and limitations around social support.^37^, ^40^ Pregnancy during adolescence was globally suggested to increase personal and family financial burden, ultimately affecting future trajectory, with lasting impact often extending to the health of the future child.^26^, ^27^, ^48^, ^49^ Additionally, adverse pregnancy outcomes place risk on the pregnant individual's health long after pregnancy, and the cost of this morbidity is not well understood and remains understudied.^44^ This dimension provides further evidence of the pervasiveness of violence against women, illuminating how limitations in systems and support in many countries globally impose often irreversible opportunity costs,^21^, ^47^ costs to the family unit and bonding,^25^, ^48^ financial costs,^42^ and future health costs^25^, ^28^, ^50^ and may impact the entire pregnancy experience for the individual by increasing the level of pregnancy burden experienced.

### Inequity

The relationship between inequity and pregnancy burden was pervasive throughout this analysis, with the subdimensions of gender and hidden burden and race and ethnicity. The type and level of pregnancy burden experienced was largely influenced by extrinsic aspects of inequity.

#### Gender and Hidden Burden

Gender has historically been a minoritizing construct globally, with continued discrimination acting as a contributor to pregnancy burden.^23^, ^24^ The gendered inequity of the expectations and responsibilities of a pregnant individual were repeatedly documented and paired with the idea of hidden burden, further highlighting the reliance of many systems globally on women as inherent solutions for unmet needs.^18^, ^20^, ^21^, ^22^, ^34^, ^39^, ^47^ Aspects of hidden burden included the multidimensional expectations of pregnancy and motherhood, the work of pregnancy, and limitations around societal support.^20^, ^21^, ^22^, ^23^, ^24^, ^25^, ^26^, ^27^, ^30^, ^31^, ^32^, ^33^, ^34^, ^36^, ^37^, ^38^, ^40^, ^41^, ^42^, ^43^, ^45^, ^46^, ^47^, ^48^, ^50^, ^52^, ^53^, ^54^ The complexity of hidden burden in pregnancy was emphasized and extended to include costs,^20^, ^21^, ^35^, ^36^, ^41^, ^52^ societal burden,^23^, ^37^ physiologic and physical burden,^18^, ^19^, ^23^, ^24^, ^29^, ^38^, ^41^, ^44^, ^46^, ^47^ emotional and mental burden,^21^, ^23^, ^24^, ^38^, ^40^, ^41^, ^42^, ^47^, ^48^, ^55^ and unacknowledged burden related to or impacted by age,^26^, ^27^, ^39^, ^48^, ^49^ health and years of life lost,^27^ care access,^24^, ^42^ time commitments,^20^, ^26^, ^38^, ^47^, ^54^ social and health inequities,^21^, ^22^, ^34^, ^45^, ^46^ and opportunity^47^, ^54^ (see Table 2).

Individuals who had given birth were described as dynamic case managers for their family, but their needs were often unacknowledged.^20^ The expectations and responsibilities of pregnancy and parenthood were not lessened for individuals with high‐demand professional responsibilities^34^, ^47^ or for those attempting to navigate social systems for resources to survive.^21^ Financial instability increased risk for poor health outcomes,^21^ whereas unplanned fertility increased early mortality later in life for individuals with higher income and education levels.^39^

#### Race and Ethnicity

Further compounding gender inequity is racial inequity, which is demonstrated in the significant burden experienced by non‐Hispanic Black pregnant individuals in the United States, and disparities in maternal health outcomes, including mortality.^24^, ^37^, ^42^ Non‐Hispanic, Black pregnant individuals in the United States experience at least double the rate of mortality as their White counterparts, independent of income or education status.^24^ The additional risk of racial discrimination alone further highlights the depth of systematic discrimination and increased burden, which appears to impact maternal health outcomes.^21^, ^23^, ^24^ Pregnancy burden may not always be visible or explicit, but influences of inequity evidences the potential to impact well‐being and health throughout the life course.

### Social Support

This dimension is significant in the experience of extrinsic pregnancy burden, encompassing 2 subdimensions including the social determinants of health (SDH) and family and other support systems.

#### Social Determinants of Health

SDH are factors that comprise an individual's life and are widely impacted by systems and structures that may positively or negatively influence outcomes.^35^, ^46^ The SDH and support from social safety net and health care systems thus influence the experience of pregnancy burden. Inability to access and navigate social safety net and health care systems creates a barrier to improving one's circumstances in pregnancy and further increases pregnancy burden due to financial, housing, childcare, and other stressors.^20^, ^21^ Pregnant and postpartum individuals described navigating unrealistic expectations of systems and the impact of SDH factors.^21^ Pregnancy and postpartum were often described as periods that contributed to further housing and job instability and necessitated extra support from social safety net and health care systems.^21^

#### Family and Other Support Systems

Family and other support systems appeared to be instrumental in moderating and managing pregnancy burden.^24^, ^40^, ^41^, ^53^, ^56^ The impact of spousal support was monumental globally and frequently cited as a determining factor between personal success for the pregnant or postpartum individual versus layered and unmanageable stress.^40^, ^47^ Individuals with strong positive family support systems experienced improved adjustment to maternal role attainment, with decreased risk for adverse outcomes.^40^ Social support also provided access to safe childcare, which often facilitated stable income in studies from countries of high‐income status.^21^, ^34^, ^47^

Contrary to the positive effects of support systems, in the Global South, authors reported pregnancy was often pressured by family or a spouse and increased burden related to conception and conflicting personal desires.^51^ In the Global North, dissatisfaction in spousal relationships and lack of family support increased the risk of pregnancy and postpartum complications, led to more sick days, and increased hospital and care expenditure, further displaying the detrimental impact of weak social support.^38^, ^40^, ^47^ An inverse relationship was noted between distress, depression, and anxiety and level of perceived social support.^40^ Although social support was frequently cited as protective, intimate partner violence risk increased during pregnancy, indicating a need to fully evaluate social support situations.^36^, ^55^ Pregnancy burden may be assumed to end after birth, but this was quite contrary to documented lived experience, which displayed evidence of increasing burden even after the pregnancy ended.

### Assumptions

Two main assumptions were noted throughout the dimensional analysis. The first was that burdensome aspects of pregnancy fell on the pregnant individual to manage and accommodate. An example of this is unavoidable intrinsic physical burden, accompanied by the need to navigate health systems (appointment availability, visitor policies, locations, etc) to attend recommended care.^20^, ^21^, ^22^ This is notably accompanied by limited resources to provide support for extrinsic stressors such as childcare, transportation, and other needs.^20^, ^21^, ^22^ These examples all additionally extend to effects on employment, career, and time off, with potentially negative consequences on health if care is missed versus financial or professional advancement consequences if employment is impacted.^20^, ^21^, ^22^, ^34^, ^47^, ^53^, ^56^

Second, although external factors may significantly influence pregnancy burden experienced by the individual, maternal health expenditure (cost of care, unintended pregnancy cost, insurance cost, etc) has been viewed as continually increasing and burdensome to systems.^21^, ^22^, ^37^, ^52^, ^53^, ^54^ Examples include cost comparison between pregnant and nonpregnant individuals, illuminating the substantial difference in expenditure required during pregnancy.^52^ Complications and unplanned pregnancy have also been highlighted as an additional financial burden and a cost to public systems,^37^, ^52^ yet financial burden notably affects the pregnant individual and may contribute to stress, factors influencing physical compensation, and care‐seeking behaviors.^20^, ^21^, ^22^, ^34^, ^47^, ^53^, ^56^

### Proposed Definition

Based on the reviewed literature, the following theoretical definition of pregnancy burden has been proposed in the context of maternal health care. *Pregnancy burden* is the increased intrinsic and extrinsic demand and compromised capacity experienced by the pregnant individual, throughout any part of their life, influenced by personal, contextual, societal, and structural factors with short‐ and long‐term consequences.

## DISCUSSION

This review has described complex and multifaceted aspects of pregnancy burden, which summarize the idea that every pregnancy is accompanied by some level of burden that can vary between individuals. A baseline level of pregnancy burden appears to be unavoidable, with intrinsic cardiovascular changes causing physiologic burden in all pregnancies.^18^, ^19^ Exploring and defining the concept of pregnancy burden has the potential to provide insight into how burden manifests during pregnancy and inform how it is addressed to improve the entire experience. Examining pregnancy burden through a holistic lens offers the opportunity to value pregnancy in its entirety and foster deeper insight into a topic that has largely been ignored. Literature on pregnancy stress–related terms has focused on pregnancy outcomes perceived to be caused by distress, more specifically fetal outcomes, rather than the comprehensive experience and needs of the pregnant individual.^57^, ^58^

Studies since 1936 do, however, expand the concept of pregnancy burden past physiologic burden and, together, propose the idea that this burden may impact quality of life and well‐being; long‐term health related to noncommunicable diseases such as hypertension, diabetes mellitus, stroke, cardiovascular disease, and other morbidities; and socioeconomic status and poverty, and emphasize that measuring this burden is necessary to demonstrate the magnitude of pregnancy burden on outcomes. The concept of pregnancy burden additionally emerged inductively in qualitative studies,^26^, ^51^ further supporting the need to advance research understanding this phenomenon. Birth trauma and fear of birth, among other related concepts, are suspected to significantly impact pregnancy experiences, further perpetuating burden. Although pregnant individuals have described pregnancy burden throughout the literature for many years, the theme of this burden remaining hidden alludes to a possible larger societal perception of the work of pregnancy.^27^

The 5 key domains for the SDH include economic stability, education access and quality, health care access and quality, neighborhood and built environment, and social and community context.^59^ Recent literature has expanded to include structural determinants as well, to account for the relationship between inequities and racism.^35^, ^60^ Limitations in access to comprehensive reproductive health services, including family planning and preconception care, safe abortion services, prenatal, birth, and postpartum care, remain evident through this analysis as an issue of equity that both impacts and is impacted by individual access and resources and would benefit from further research to explore how these limitations impose burden.

Although types of burden experienced may vary between individuals of varying health background, race, ethnicity, socioeconomic status, or education level, this analysis shows that factors contributing to level of pregnancy burden are frequently modifiable. Findings from this analysis also highlight a possible connection between level of pregnancy burden experienced and structural and SDH, discrimination, and racism. Pregnancy burden largely falls on pregnant individuals in most countries globally but is the concern of everyone, with lasting effects. Addressing pregnancy burden's modifiable contributors will likely require societies globally to recognize burden and invest in adequate resources to support all dimensions of the individual's life.

Development of the concept of pregnancy burden may allow for advancement of clinical care, social systems, and policy to support patients and alleviate burden to improve the experience of pregnancy. Appreciation of the entire pregnancy, inclusive of burden, is an opportunity for society to respect the total reality that the pregnant individual experiences. As the current base of existing research on pregnancy burden is limited, the body of evidence warrants further attention and development to better understand what pregnancy burden is, its contributors, its relationship to outcomes, and modifiable factors.

### Strengths and Limitations

There are several limitations to this concept analysis. The results in this article have been extrapolated from studies investigating various aspects of pregnancy that have shown burden. Rather than include synonyms for the broader concept of burden, the search terms used within this review were limited as an attempt to determine how pregnancy and burden have been previously conceptualized together. Through the review and analysis, it became clear that pregnancy burden exists on a continuum. Although including terms such as *difficulty*, *stress*, *challenges*, or *concerns* with *pregnancy* may have expanded results, these terms were not included.

An additional limitation is that prior literature has largely focused on self‐perceived and chronic burdens and has not explored burden as it relates to pregnancy.^61^, ^62^, ^63^ This is further compounded by limited research seeking to explore the perspective or experience of a pregnant person.^1^, ^64^ Pregnancy stress literature uses many similar terms that are undefined, with no standardized tools or measurement, and a strong focus on fetal outcomes.^58^ Negative or emotionally laden aspects or feelings during pregnancy, outside of medicalization or a diagnosed mental illness, have also rarely been examined.^26^ This analysis revealed that a vast number of tools are being used to explore dimensions and subdimensions of pregnancy burden independently but do not account for the comprehensive experience. Further instrument review is necessary, as this imposed a limitation on what was available for review and previous measurement. Pregnancy burden is a fruitful area for mixed‐methods research, using community‐informed methods.

Although there are several limitations to this integrative review and concept analysis, there are also strengths. The first is the importance of this work and the significance of the need that has been identified through this analysis. The literature review process was rigorous, and the literature search strategy process was informed by an expert academic librarian. This review unintentionally resulted in findings from 19 countries and provided a global review of pregnancy burden in the current literature. Additionally, all steps of the dimensional concept analysis method were used, which further increased strength and rigor of the analysis.

## CONCLUSION

The findings of this analysis show that pregnancy burden exists but is understudied and has not had a prior definition. Although general themes of pregnancy burden are widely present in the literature, pregnancy burden as a unique and individualized experience has not been widely studied. Prior to this analysis, a comprehensive definition had not been proposed, and research has not attempted to quantify or qualify it. The dimensional analysis method developed by Caron and Bowers (2000)^16^ was used to identify descriptive phrases and attributes to guide in the discovery of the conceptual dimensions, clarify involved perspectives, and develop the literature‐derived theoretical definition that has been proposed for pregnancy burden. This proposed definition may support health care professionals, researchers, and the included perspectives from this analysis to foster discussions of pregnancy from an intentional, holistic viewpoint while simultaneously creating a uniform use of terminology. This analysis revealed the need for further exploration of pregnancy burden to explore its impact on the pregnant person and family, the role of social and structural determinants of health on pregnancy burden, and factors that can be modified to decrease burden during pregnancy. Better understanding and appreciation of the holistic journey of pregnancy, inclusive of burden, has the potential to improve the pregnancy experience and potentially impact birth‐related outcomes.

## CONFLICTS OF INTEREST

The authors have no conflicts of interest to disclose.

## Supporting information




**Appendix S1**. Bibliographic Literature Searching
