# Supplementary material for: Pregnancy Burden: An Integrative Review and Dimensional Analysis of Pregnancy's Hidden Challenges
Source: J Midwifery Womens Health. 2025 May 22;70(5):717–32. doi: 10.1111/jmwh.13759 (PMC12529887; doi:10.1111/jmwh.13759)
Supplement: Supplementary file 1 — Appendix S1. Bibliographic Literature Searching [file JMWH-70-717-s001.docx]

**ONLINE APPENDICES:**

## ***Electronic Supplementary Material Appendix 1***

Bibliographic Literature searching

| **Database** | **Hits** |
| --- | --- |
| CINAHL Plus with Full Text | 1080 |
| PsycINFO | 1179 |
| PubMed | 2599 |
| Scopus | 1151 |
| Total | 6009 |
| - duplicates | 1895 |
| Unique studies to screen | 4114 |

Database: CINAHL Plus with Full Text

Host: EBSCOhost

Data Parameters: 1937 to Present

Date Searched: August 1, 2023

Searcher: Rebecca Billings

Search Strategy:

| **#** | **Searches** | **Results** |
| --- | --- | --- |
| 1 | ((MH "Pregnancy+" OR MH "Fertility+" OR TI fertil* OR AB fertil* OR MH "Family Planning+" OR TI "family planning" OR AB "family planning" OR TI maternal OR AB maternal OR MH "Infertility+" OR TI infertil* OR AB infertil* OR MH "Postnatal Period+" OR TI perinatal OR AB perinatal OR TI postnatal OR AB postnatal OR TI postpartum OR AB postpartum OR TI sterility OR AB sterility OR TI subfertility OR AB subfertility OR TI childbearing OR AB childbearing OR MH "Reproductive Behavior" OR MH "Reproductive Health" OR TI reproductive OR AB reproductive OR TI reproduction OR AB reproduction) | 385687 |
| 2 | TI burden* | 21456 |
| 3 | #1 AND #2 | 737 |
| 4 | (pregnan* N5 burden*) | 436 |
| 5 | #3 OR #4 | 1080 |

Notes: Build search in basic search box and combine queries in Search History.

Database: PsycINFO

Host: ProQuest

Data Parameters: 1806 to Present

Date Searched: August 1, 2023

Searcher: Rebecca Billings

Search Strategy:

| **#** | **Searches** | **Results** |
| --- | --- | --- |
| 1 | (MAINSUBJECT.EXACT.EXPLODE("Pregnancy") OR pregnan*) | 89675 |
| 2 | AND burden* | 60046 |
| 3 | #1 AND #2 | 1179 |

Notes: Build search in the Advanced search command line.

Database: PubMed

Host: National Library of Medicine (<http://www.ncbi.nlm.nih.gov/pubmed>)

Data Parameters: 1946 to Present

Date Searched: August 1, 2023

Searcher: Rebecca Billings                      

Search Strategy:

| **#** | **Searches** | **Results** |
| --- | --- | --- |
| 1 | (("Pregnancy"[Mesh] OR "Fertility"[Mesh] OR fertil*[tiab] OR "family planning"[tiab] OR maternal[tiab] OR "Infertility"[Mesh] OR infertil*[tiab] OR "Perinatal Care"[Mesh] OR "Postpartum Period"[Mesh] OR perinatal[tiab] OR postnatal[tiab] OR postpartum[tiab] OR sterility[tiab] OR subfertility[tiab] OR childbearing[tiab] OR "Reproductive Behavior"[Mesh] OR reproductive[tiab] OR reproduction[tiab]) AND burden*[ti]) | 1748992 |
| 2 | burden*[ti] | 45064 |
| 3 | #1 AND #2 | 1858 |
| 4 | ("pregnancy burden"[Title/Abstract:~5]) | 657 |
| 5 | ("pregnancy burdens"[Title/Abstract:~5]) | 56 |
| 6 | ("pregnancy burdensome"[Title/Abstract:~5]) | 13 |
| 7 | ("pregnant burden"[Title/Abstract:~5]) | 300 |
| 8 | ("pregnant burdens"[Title/Abstract:~5]) | 16 |
| 9 | ("pregnant burdensome"[Title/Abstract:~5]) | 4 |
| 10 | #4 OR #5 OR #6 OR #7 OR #8 OR #9 | 1016 |
| 11 | #3 OR #10 | 2599 |

Notes: N/A

Database: Scopus

Host: Elsevier

Data Parameters: 1960 to Present

Date Searched: August 1, 2023

Searcher: Rebecca Billings                      

Search Strategy:

| **#** | **Searches** | **Results** |
| --- | --- | --- |
| 1 | (TITLE-ABS((pregnan* W/5 burden))) | 1151 |

Notes: Copy/paste search strategy in the Advanced document search.
